# The first hydrophobic region of the HPV16 E5 protein determines protein cellular location and facilitates anchorage-independent growth

**DOI:** 10.1186/1743-422X-5-30

**Published:** 2008-02-26

**Authors:** Caroline Lewis, Marta F Baro, Margarita Marques, Myriam Grüner, Angel Alonso, Ignacio G Bravo

**Affiliations:** 1 Deutsches Krebsforschungszentrum, Im Neuenheimer Feld-242, 69120 Heidelberg, Germany; 2 Universidad de León, 24071 León, Spain; 3 Experimental Molecular Evolution. Institute for Evolution and Biodiversity. Westfaelische Wilhems University Muenster, Hüfferstrasse 1, Germany

## Abstract

The human papillomavirus type 16 E5 protein (HPV16 E5) is 83 amino acids in length and contains three well-defined hydrophobic regions. The protein is expressed at very limited amounts in transfected cells and the absence of specific antibodies has strongly hampered functional analyses. To investigate the relationship between structure and function we have synthesized a codon-adapted version of the gene (hE5) and prepared a series of N-terminal and C-terminal deletions. Immunofluorescence analyses show colocaliation of the protein with calnexin, an ER marker, EEA-1, an early endosomes marker, and Lamp-2, a lysosomal marker. No major colocalization was found between hE5 and the Golgi marker 58 K. Whereas deletions at the C-terminal end of the protein do not greatly alter the localisation pattern, deletion of the first hydrophobic region results in loss of colocalisation with the ER, early endosomes and lysosomes. Further, we show that while the complete E5 protein confers to HaCaT cells the property to grow in an anchorage-independent manner, deletion of the first hydrophobic region results in loss of growth in soft agar. We conclude that the first hydrophobic region of the E5 protein largely determines the biological properties of the viral protein.

## Background

Certain papillomaviruses (PVs) present a coding region between the E2 and L2 open reading frames. The proteins herein encoded are usually named E5, although they can be classified into four different types according to their phylogeny and to their correlation with abnormal growth [1]. HPV16 E5 is the prototype of the E5-α group, and is the most investigated E5 protein. The HPV16 E5 is 83 amino acids in length and mainly localises in the Golgi apparatus and the endoplasmic reticulum [2-4].

The cellular effects associated to the expression of HPV16E5 are multiple [5]. Experimental work has demonstrated that HPV16 E5 binds the 16 K proteolipid subunit of the membrane proton pump, although this binding does not appear to be responsible for the E5-mediated epidermal growth factor receptor (EGFR) over-activation [4,6,7]. The binding region has been mapped to amino acids 54 to 78 [8] or to amino acids 41 to 54 of the E5 protein [7], and in both cases this binding was unrelated to the overactivation of the EGFR. In addition, data have been published showing that HPV16 E5 binds the platelet-derived growth factor as well as the EGF receptors, although contradictory results have been reported [9,10]. Regarding the interaction of the E5 proteins with the immune system, it has been proposed that its expression blocks the transport of the major histocompatibility complexes to the cell surface [11,12], either by direct interaction with the heavy chain of the human leucocitary antigen molecule [13] or indirectly via interaction with calnexin [14]. Finally, it has been suggested that many of the multiple and disparate effects associated to E5 could eventually arise from modifications in membrane composition and dynamics subsequent to protein expression [15].

The strong hydrophobicity of the protein and the absence of valuable specific antibodies have hampered E5 research. Most of the experimental work with the protein has been performed using a tagged gene and antibodies to the ligated epitope. In addition, the rare use of the viral codons by mammalian cells makes expression of the protein very weak, thus difficulting the identification of the epitope-tagged proteins as well as the analysis of its putative biological effects. In most cases, expression has been limited to the demonstration of the corresponding RNA [16]. For these reasons, the group led by Schlegel synthesized an E5 gene adapting the codon usage to that found in mammalian cells [17]. Using this "codonadapted" gene they observed a strong expression of the protein and found that the protein mainly localised in the endoplasmic reticulum [17]. This contrasts with other published results using the "wild-type" codons showing a clear colocalisation of the E5 protein with markers specific for the Golgi apparatus [2].

Hydropathic analysis of the protein reveals three hydrophobic regions [1], the first being considered to be the putative transmembrane region, although no experimental work has so far demonstrated this point (see [18] for BPV1 E5). At the HPV16 E5 C-terminal end, a short hydrophilic tract seems to be involved in protein-protein associations and also to be responsible for the activity of the viral protein towards the EGF receptor [7,8].

We have performed a series of experiments aiming to analyse the correlation between the primary structure of the protein and its cellular localisation and biological function. For this, deletions encompassing fragments of the N-terminal or C-terminal ends of the E5 protein were constructed, and the cellular localisation and biological effects of these modified genes were analysed. Our results indicate that the first hydrophobic region of HPV16 E5 determines the subcellular localisation of the protein, despite no canonic localisation signal can be identified. Finally, cells transformed with the full-length HPV16 E5 gene were able to grow in an anchorage-independent manner, whereas transformants lacking the first hydrophobic domain do not grow in soft agar.

## 3.1 Results

### 3.1. Preparation of recombinants and expression of the proteins

**Figure 1:**
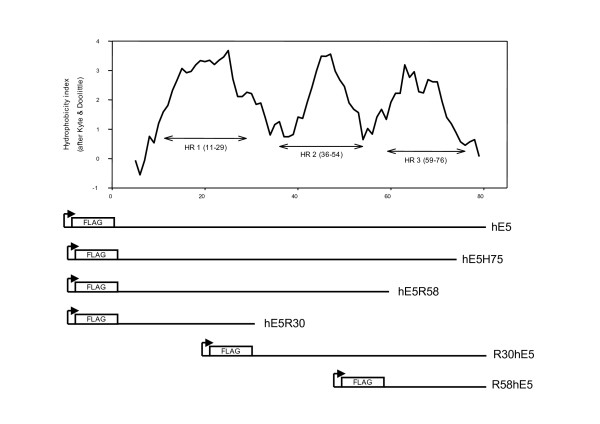
**A) Hydropathic analysis of the HPV16 E5α protein using a window of 9 amino acids and the Kyte&Doolittle index.** HR: hydrophobic region. B) Schematic representation of the mutants employed in our experiments. Sequences were cloned into the Eco Rl-Bam Hl restriction sites of the vector pFlag-CMV4. In all cases the start methionine amino acid was deleted. All recombinants were confirmed by sequencing.

A recombinant containing the human codon-adapted sequence of HPV16 E5 (hE5), an α-type E5 protein [1] (EF463082) was cloned into the vector pFlag-CMV4 (Sigma). Cloning was performed deleting the methionine start codon of the viral protein and ligating the rest of the E5 gene the vector Flag epitope. The HPV16 E5 protein is 83 amino acids long, with three well-defined hydrophobic regions (Fig. 1). In an initial series of experiments, we produced series of deletions starting at the first viral amino acid after methionine and with stop codons at amino acids histidine 75 (hE5H75), arginine 58 (hE5R58), or arginine 30 (hE5R30). N-terminal deletions were prepared starting at amino acids 30 (R30hE5) or 58 (R58hE5) and ending at amino acid 83.

**Figure 2:**
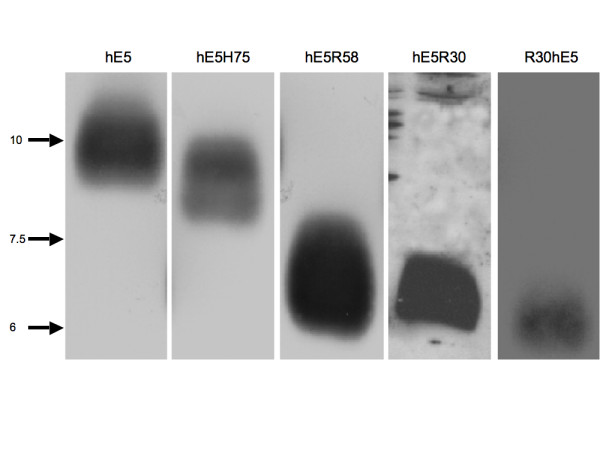
**Expression of the codon-adapted version of HPV16 E5α and corresponding deletions.** HEK- 293T cells were transfected with the pFlag-pCMV recombinants and 24 hours thereafter SDS extracts were prepared. Proteins were separated by acrylamide gel electrophoresis and blotted with antibodiesagainst the Flag-epitope.

To analyse expression of the protein mutants, HEK-293T cells were transfected with pFlag-CMV4 recombinants and 24 hours later protein extracts were prepared. The proteins were separated by polyacrylamide gel electrophoresis and blotted with antiflag antibodies. As shown in Fig. 2, all recombinants were expressed in HEK-293Tcells albeit with different efficiencies. Whereas deletion hE5R58 was strongly expressed, deletion R30hE5 was expressed at much lower levels. No protein could be observed using deletion R58hE5. Results concerning this mutant will therefore not be considered in this manuscript.

These results were reproducible when using different DNA batches and performing the transfections on different days, suggesting that the differences observed among the deletions are of intrinsic nature and not experimental artefacts.

### 3.2. Cellular localisation of wild-type and mutant E5α proteins

It has been reported that, when expressed from a codon-adapted gene, the E5α protein is mostly localised at the endoplasmic reticulum [17]. To identify the cellular compartment where the deletions were located, we used immunofluorescence colocalisation experiments via specific antibodies against markers for the Golgi apparatus (58 K), the early endosomes (EEA-1), the endoplasmic reticulum (calnexin), and the lysosomes (LAMP-2).

**Figure 3:**
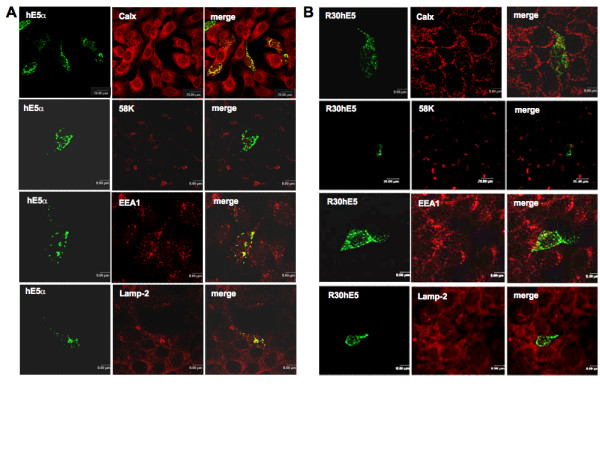
**Cellular localisation in of hE5 protein and deletions expressed from codon-adapted genes.** HaCaT cells cells were transfected with the complete gene or with the R30hE5 deletion. Colocalisation with markers specific for endoplasmic reticulum (Calnexin) Golgi (58 K), early endosomes (EEA1) or lysosomes (Lamp-2) was analysed by double immunofluorescence.

**Figure 4:**
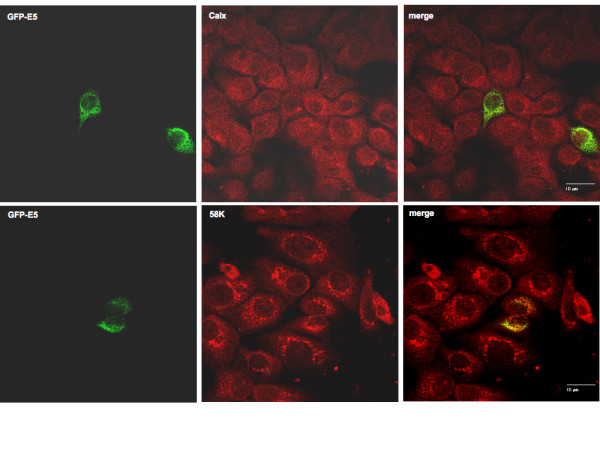
**Cellular localisation in HaCaT cells of GFP-E5 protein expressed from wild-type viral gene.** Colocalisation with markers specific for endoplasmic reticulum (Calnexin) and Golgi (58 k) was analysed by double immunofluorescence.

In full-length transfectants carrying the codon-adapted version of the gene a strong colocalisation with calnexin was found (Fig. 3A), consistenly with previous reports [14]. This contrasts with the distribution detected when the full-length gene carrying the viral codons was transfected. In this case almost no colocalisation with calnexin was observed (Fig. 4), also consistently with previous reports [2]. Further, all Cterminal deletions showed a strong colocalisation with calnexin. Surprisingly, no colocalisation was found between the N-terminal mutant R30hE5 and calnexin, indicating that the mutant protein is not localised in the ER (Fig. 3B).

**Figure 5:**
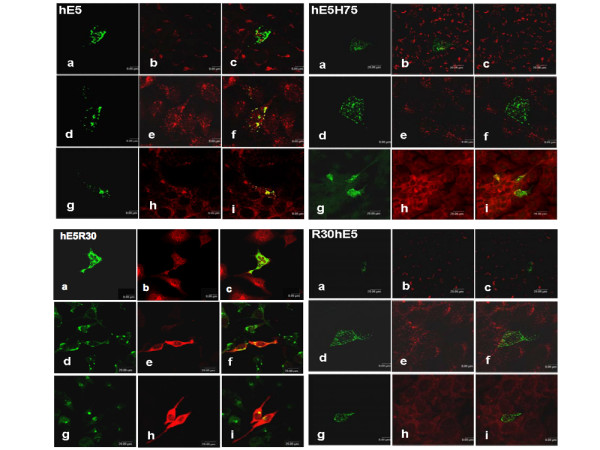
**Cellular localisation of hE5 protein and deletions expressed from codon-adapted genes.** HaCaT cells were transfected with the complete gene or with the deletions hE5H75, hE5R30 or R30hE5. Colocalization with markers specific for Golgi (58 K, pictures b, c), early endosomes (EEA1, pictures e, f) or lysosomes (Lamp-1, pictures h, i) was analysed by double immunofluorescence. Overlays are shown in pictures c, f and I, respectively.

Our immunofluorescence analyses further demonstrate that neither the codonadapted full-length gene nor any of the codon-adapted deletion mutants colocalised with the 58 K marker for the trans-Golgi (Fig. 3A, B, Fig. 5). This again contrasts with the picture observed when the full-length viral gene was transfected, where colocalisation of E5 and 58 K was observed (Fig. 4).

Clear colocalisation with EEA-1 was found for both the full-length gene and the Cterminal deletions, indicating the presence of the protein in the early endosomes. Finally, a strong colocalisation of the wild type protein and deletion hE5H75 with Lamp-2 was observed, suggesting that degradation of the protein -or at least of this particular deletion- takes place in the lysosomes (Fig. 5). Interestingly, no colocalisation with any of the markers used could be demonstrated for mutant R30hE5 (Fig. 3B), despite the clear expression of the protein as demonstrated in the Western blots (see Fig. 2). The immunofluorescence pictures suggest that this mutant protein is associated to some membranous structures and not randomly distributed in the cytosol (Fig. 3B). A summary of the immunofluorescence data is shown in table 1.

**Table 1:** Colocalisation in HaCaT cells of E5 proteins with markers specific for ER, trans-Golgi network, early endosomes, or lysosomes.

		Colocalisation with		
	
	Calnexin (endoplasmic reticulum)	58 k (trans-Golgi network)	EEA1 (early endosomes)	Lamp-1 (lysosomes)
Construct				
hE5	++	-	+++	+
hE5H75	+/-	-	+/-	+
hE5R58	+	-	++	+
hE5R30	++	-	++	+
R30hE5	-	-	- (dubious +/-)	-

Relative intensity of colocalisation is shown by symbols: -, no colocalisation; -/+, occasional colocalisation; +, weak colocalisation; +++, strong colocalisation.

### 3.3. Growth in soft agar

**Figure 6:**
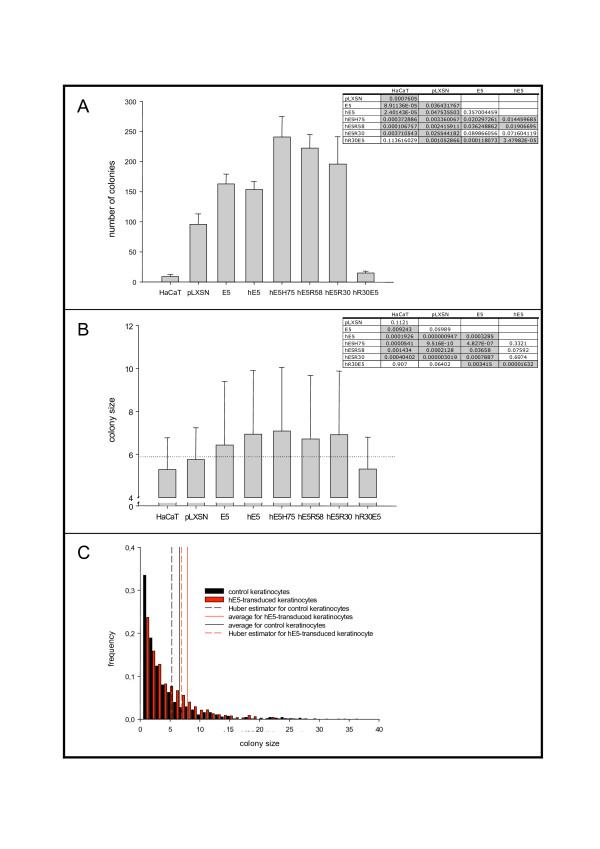
**Growth of transduced HaCaT cells in soft agar.** Cells were plated on soft agar and overlayed with DMEM containing 50 ng/mL EGF. After 21 days growth dishes were stained with Cristal violet and the colony number (A) or colony size (B, C) was calculated. A) average values for the number of colonies generated in each different transfectant. Error bar encompass 95% confidence interval of themean. Inset A) P-values for a one-tail Student's t-test. Values with statistically significant differences have been shadowed. B) values for the Huber central estimator for the colony size -arbitrary units- in each different transfectant. Error bar encompass the median absolute deviation. The vertical scale has been broken in the interval 0–4 since the smallest colony size detectable with the hardware/software employed was four units. Dashed line marks the 95% confidence value for the colony size in the HaCaT control cell lines. Inset B) P-values for a Wilcoxon-Mann-Whitney test; H0 = median values of both populations are similar. Values with statistically significant differences have been shadowed. C) Example showing the histograms for distribution of colony size in control HaCaT cells -black- and in hE5 transfectants -red. Continuous lines mark the corresponding values for the average of the population. Dashed lines mark the corresponding values for the Huber central estimators.

Expression of HPV16-E5 in primary keratinocytes from a codon-adapted gene does not result in alterations of ligand-mediated EGFR phosphorylation, PI 3-K or c-Src activation but promotes anchorage-independent growth in soft agar [19]. Thus, we chose keratinocyte growth in soft agar as a read-out for the biological activity of the protein and the corresponding deletions. HaCaT cells were transduced with retroviruses carrying the complete gene or the different deletions, plated onto soft agar, and 21 days later colony number and size were scored. The values obtained for colony size followed a exponential decrease-like distribution, but could not be properly fitted to any reasonably simple function (an example is shown in Fig. 6C). We have therefore addresed the statistic comparisons by means of robust estimators (Huber estimator for central tendency and median absolute deviation for dispersion) and by means of robust comparisons (Wilcoxon-Mann-Withney test).

Non-transduced HaCaT cells were only marginally able to grow under non-adhesive conditions, confirming published reports [20]. An interesting observation in this experiment was that both genes, codon-adapted and original viral codons, stimulated growth in soft agar to similar extents, despite the differences in protein expression and in subcellular localisation. As shown in Fig. 6, the full-length genes as well as the deletions investigated, with the exception of deletion R30hE5, increased the number of colonies and the colony size of keratinocytes growing in soft agar. The effect of the empty vector on colony formation was noteworthy. As shown in Fig. 6A, HaCaT cells transduced with the empty pLXSN retroviral vector were able to form colonies in softagar, albeit the size did not differ from the untransduced controls (p = 0.1121, Wilcoxon-Mann-Whitney test).

Analysis of the colony size revealed that cells transduced with a codon-adapted E5 gene were larger than cells transduced with the wild-type gene (p = 3.285e-3, Wilcoxon-Mann-Whitney test) (Fig. 6B). All three codon-adapted C-terminal deletions induced also the growth of larger colonies than the wild-type gene, and showed no statistical significant differences in size compared to the full-length codon-adapted transductants. Finally, the few colonies grown after transduction with the N-terminal deletion hR30E5 were did not differ in size from control HaCaT cells (p = 0.907, Wilcoxon-Mann-Whitney test).

In summary, keratinocytes transduced with constructs encompassing the first hydrophobic region of HPV16 E5 showed increased growth under anchorageindependent conditions. Furthermore, full-length codon-adapted transductants gave rise to more colonies and to larger colonies than full-length wild-type HPV16 E5 transductants.

## 4. Discussion

In this communication we present experimental evidence demonstrating expression in HEK-293T cells of a codon-adapted version of the HPV16 E5α protein, as well as of a series of N- or C-terminal deletions. Expression of mutant protein hR58E5 could not be demonstrated in western blots. Whether the failure to identify expression of this mutant is due to inherent protein instability is unknown. Immunofluorescence experiments with markers specific for different cellular compartments show colocalisation of HPV16 hE5α with calnexin, indicating that the protein is mainly localised at the endoplasmic reticulum. This finding is in agreement with previous results obtained also using codon-adapted versions of the E5 gene [14,17]. Interestingly, these results contrast with published reports showing the protein mainly associated with the Golgi apparatus [2,3]. It must be noted however that the initial reports refer to genes encoding the original, non-human-adapted viral codons. A possible explanation for this difference may lie in the large amounts of E5 protein synthesised from the codon-adapted genes, which will probably accumulate in the ER with the cell being unable to further transport the protein to the Golgi compartment. Similar remarkable shifts in cellular localisation and/or function have also been reported for the L1 [21], E6 [22] and E7 [23] proteins from different PVs after humanisation of the codon usage. Experimental differences between the effects of the expression of wild-type and humanised PV genes highlights again the physiological importance of the biased codon usage in PVs [24,25]. The colocalisation demonstrated for E5α with the early endosomal marker EEA-1 is most interesting since a physical association between HPV16 E5α and the 16 K subunit of the proton ATPase has already been demonstrated. This association has been made responsible for the modulation of the internal pH value of the endosomes [4,6]. In addition, the presence of HPV16 E5α in early endosomes is in agreement with the functional observation of an E5-dependent pH alteration in endosomes, as previously described [17,26].

The observation that the presence of the first hydrophobic region is necessary for localisation of the protein to the ER, early endosomes or lysosomes is noteworthy, mainly because of the lack of a canonical signal peptide. Deletion of the first 30 amino acids (recombinant R30hE5) results in changes in both localisation pattern and biological effects of the protein. This mutant showed no loss colocalisation of the Flag epitope with 58 K, calnexin, EEA-1 or Lamp-2. Moreover, in the anchorageindependent growth experiments this mutant strongly inhibited colony growth and the size of the few growing colonies was clearly reduced in comparison with that of the full length gene. Further, the immunofluorescence pictures showed a punctuated distribution for this mutant, suggesting that the protein was associated with some kind of vesicular structure, although we were not able to identifiy it.

The results shown in this communication demonstrate that the expression of theHPV16 E5 protein increases the number and the size of HaCaT cell colonies growing in soft-agar. The results were the same for both the wild-type version of the E5 gene and for a codon-optimised version, despite strong differences in expression intensity between these two genes. This suggests that the amount of expressed E5 protein is not decisive for colony number but determines the number of cells and therefore the colony size (see Fig. 6). The data here detailed represent the first quantitative description of the ability of HPV16 E5 to promote growth in soft agar. A previous qualitative description in the same sense had been provided by Suprynowicz and coworkers, in primary keratinocytes [19]. The effects of HPV16 E5 on uncontrolled cell growth and malignisation seem therefore to be multiple. Thus, although an increased expression of the protein seems to shorten the in vitro life span of primary keratinocytes [17], the expression of HPV16 E5 allows human keratinocytes to grow in soft agar ([19] and the present paper). Finally, as a correlate at the organismic level, the expression of HPV16 E5 in transgenic mice leads to the development of endophytic papillomas, precursors to carcinomas, and contributes to the promotion and progression stages of carcinogenesis [27].

Regarding the dissection of the differential implication of the three transmembrane domains of HPV16 E5 in the biological effects of the protein, our finding that Nterminal deletion mutant R30hE5 was unable to promote growth in soft-agar is interesting since this mutant did not show colocalisation with any of the markers used in our immunofluorescence experiments. The molecular mechanisms responsible for this effect are unknown. Computer analysis of the protein identifies the first hydrophobic region of HPV16 E5 as a putative trans-membrane segment [1,28]. It may be speculated that interactions of with other membrane proteins determine the biological activity of the viral protein. This is further supported by recently published work showing that E5 interacts in the ER with calnexin through the first hydrophobic segment [14]. This interaction results in retention of the HLA class I molecules in the Golgi apparatus, with concomitant down-regulation of its plasma membrane expression [11,13,14].

Thus, we can conclude that the first 30 amino acids of the HPV16 E5α protein play a crucial role in the biological properties of the protein. This region determines cellular localisation of the protein, binding to the chaperone calnexin, and anchorageindependent growth in a human keratinocyte cell line.

## Materials and methods

### Recombinants

The nucleotide sequence of the codon-adapted HPV16 E5 gene was synthesized *in vitro *adapting the wild type codons to the human codon usage (GeneArt, Regensburg), and the corresponding sequence has been deposited in GenBank under EF463082. The full-length gene and deletions encompassing different fragments of the protein were cloned into the Eco RI-Bam Hl sites of recombinant pLXSN (Fig. 1). For immunofluorescence and western blot experiments, hE5 sequences were cloned into the vector pFlag-CMV4. For comparing subcellular distribution of the codon-adapted genes and the original viral sequence, a GFP-E5 fusion recombinant was synthesized by ligating the E5 wild-type coding region to the C-terminal end of the green fluorescence protein gene of the pEGFP vector [3].

#### 2.2. Cell culture, transfections, and transductions

HaCaT cells, an immortalized human keratinocyte cell line, were cultured in DMEM supplemented with 10 % FCS and antibiotics. Transfections with the pCMV4 recombinants were performed using Lipofectamine 2000 as indicated by the manufacturer. 24–48 hours after transfection the cells were fixed in 4% paraformaldehyde. Double immunofluorescence was performed using polyclonal antibodies against the Flag tag, and monoclonal antibodies against the 58 K Golgi protein (Sigma-Aldrich), endoplasmatic reticulum marker calnexin (Santa Cruz), the lysosomal marker LAMP-2 (antibody H4B4, University of Ohio) and the early endosomal marker EEA-1 (Transduction Laboratories). Pictures were obtained using a confocal microscope LEICA DMRBE.

For the analysis of protein expression, recombinants were transfected into HEK-293T cells. 24 hours after transfection protein extracts were prepared and immunobloted with antibodies to the Flag epitope. Reacting bands were revealed with ECL.

HaCaT cells were transduced with retroviruses as described [29]. In brief, retroviruses were generated in Phoenix cells transfected with the corresponding recombinants. The cell supernatants containing the retroviruses were used to transduce HaCaT cells in the presence of polybrene. After 24 hours growth, 800 μg/mL of the selection antibiotic G-418 were added and the cells were further cultured for the desired time.

#### 2.3. Anchorage-independent growth

The growth characteristics of transduced HaCaT cells under anchorage-independent conditions were analysed by growing the cells in soft agar. 2 × 10^4^, 5 × 10^4 ^or 10^5 ^cells transduced with the corresponding retroviral constructs were suspended in 2× MEM containing 20 % FCS and mixed with the same volume of 0.7% agar at 42°C. Cells were plated onto 0.5% agar in medium and then overlayed with 200 μL of medium containing G-418 (800 μg/μL) and 50 ng/mL EGF. Each point was performed twice in triplicate. The experiment was repeated twice using different retroviral supernatants and different keratinocyte cultures. Each point thus gathers information from at least 12 agar plates. Colony number and size were scored after 3 weeks growth, using crystal violet staining and the ImageQuant software (Amersham). Due to the non-normal distribution of the colony size, the robust Huber estimator was used for central tendency. Comparison among colony size values was performed with the Wilcoxon-Mann-Whitney test, implemented in R. Comparison among number of colonies was performed with a one-side Student's t-test.

## Authors' contributions

CL performed the initial cloning, constructed the gene deletions and confocal microscopy. MFB and MM performed the soft-agar growth experiments. MG performed the retroviral subcloning. AA designed the experiment and drafted the manuscript. IGB designed the experiment, analysed the data and drafted the manuscript. All authors have agreed on the final version of the manuscript.
